# Osteonecrosis Research in Orthopedics and Traumatology: A Global Bibliometric Analysis of Publication Trends, Key Contributors, and Thematic Evolution

**DOI:** 10.1111/os.70332

**Published:** 2026-05-19

**Authors:** Guadalupe A. Pomasonco‐Olivas, Ulises J. Baldeon‐Necochea, Alvaro M. Ñaña‐Cordova, Fabriccio J. Visconti‐Lopez

**Affiliations:** ^1^ Universidad Científica del Sur Lima Peru; ^2^ CHANGE Research Working Group, Facultad de Ciencias de la Salud, Carrera de Medicina Humana. Universidad Científica del Sur Lima Perú

**Keywords:** bibliometric analysis, bone necrosis, orthopedics, osteonecrosis, traumatology

## Abstract

**Objective:**

Osteonecrosis is a multifactorial pathology of importance in the field of orthopedics and traumatology. The volume of publication and thematic evolution has not been previously analyzed. This study aimed to characterize the scientific production related to osteonecrosis in orthopedics and traumatology, identify the most influential authors, the journals with the highest impact, and the emerging trends in research.

**Methods:**

A bibliometric analysis was performed using the Web of Science Core Collection database. The bibliographic search was performed within the category “Orthopedics,” limiting to original articles up to the year 2024. Reviews and other types of non‐original publications were excluded. The analysis was performed with the statistical package Bibliometric (R) and VOSviewer 1.6.17, by manual data cleaning and analysis of co‐authorship networks, affiliations and co‐occurrence of keywords.

**Results:**

1620 articles distributed in 78 journals were identified. The year with the highest number of publications was 2024 (131 articles), with an annual growth rate of 29.25%. Michael A. Mont was the most productive author (34 articles) and Stuart Goodman the most cited (23,139 citations). China was the country with the highest number of publications (375), while the United States led in number of citations (9578 total; 26.98 per article). The most productive journals were *Journal of Orthopaedic Surgery and Research* (414) and *International Orthopaedics* (365); however, *Journal of Arthroplasty* (1733) had the highest citation impact. The most frequent keywords were “osteonecrosis,” “core decompression,” and “mesenchymal stem cell.” The most cited article was “Risk factors associated with deep surgical site infections after primary total knee arthroplasty: an analysis of 56,216 knees” with 451 citations.

**Conclusion:**

Research on osteonecrosis in orthopedics and traumatology has shown sustained growth, with an emerging focus on advanced surgical interventions and regenerative strategies. The United States and China stand out as leaders in volume and impact, respectively. This bibliometric analysis offers key tools to guide future research, strengthen international collaboration, and improve therapeutic strategies in this pathology.

## Graphical abstract

This bibliometric analysis reveals rising global interest in osteonecrosis, with emerging trends centered on joint‐preserving techniques and stem cell therapy. The evolving research landscape highlights future directions in surgical innovation and regenerative medicine.

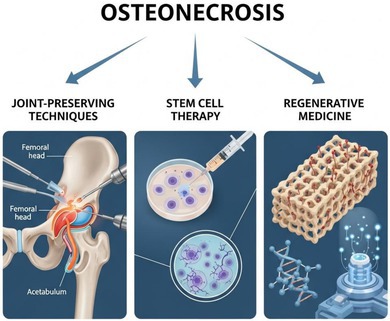

## 1 Introduction

Osteonecrosis is defined as the death of bone cells secondary to decreased or blocked blood flow to the bone [1]. This process can lead to joint involvement and subsequent deterioration of some surrounding cartilage due to the collapse of the affected bone [2]. The most common clinical manifestation of this condition is pain in the affected joint, which usually starts when the patient is carrying heavy items and then continues even at rest [3]. The most frequent locations of the lesions are in the bony points of greatest stress, such as the femoral and humeral epiphyses, carpal bones, tarsus, and knee [4]. It is estimated that approximately 20,000 new cases of osteonecrosis are detected each year in the United States. Furthermore, the total cumulative number of people affected by osteonecrosis of the femoral head is estimated to be between 300,000 and 600,000 over the last 30 years [5].

This is a pathology of multifactorial origin that is classified into two groups according to its cause: traumatic and nontraumatic [6]. In the majority of cases, traumatic‐origin injuries are caused by fractures and dislocations of the hip and knee [7]. In contrast, those of non‐traumatic origin are usually associated with systemic factors and predisposing conditions to the disease [8]. The interruption of vascular flow may be explained by a number of mechanisms, including vascular occlusions (e.g., vascular infarcts or stenosing arteritis), alterations in lipid metabolism and coagulation disorders [9]. Furthermore, it has been determined that treatment with corticoids, trauma, exposure to radiation, chemotherapy, and alcohol consumption are predisposing conditions for osteonecrosis [10]. In the older adult population, which represents the highest risk group, the main associated factors include hip fracture, solid organ transplantation and dialysis. The prognosis for recovery is more favorable in younger populations, due to the cartilage's capacity for cartilage repair [11].

The diagnosis of osteonecrosis of the femoral head requires a combination of imaging studies such as radiographs to detect bone sclerosis and magnetic resonance to identify early changes in bone vascularization [12]. Recent research has linked this pathology to COVID‐19, highlighting the need for clinical and epidemiological studies to better understand its etiology and progression [13]. In this context, bibliometric studies are essential to map the development of knowledge about osteonecrosis, identify emerging trends, and guide future research toward more effective treatments [14]. Additionally, this type of study helps to highlight gaps in knowledge and guide the development of new diagnostic and therapeutic strategies that improve clinical outcomes [15]. For these reasons, this study aimed to characterize the scientific production related to osteonecrosis in orthopedics and traumatology, identify the most influential authors, the journals with the highest impact, and the emerging trends in research.

## 2 Methods

### 2.1 Search Strategy and Eligibility Criteria

A bibliometric analysis of articles published in journals indexed under the category “Orthopedics” in the Web of Science (WOS) Core Collection was performed. The search strategy was performed using the following terms: TS = (“osteonecrosis” OR “aseptic necrosis* of bone*”). Another term for this condition is “avascular necrosis of bone”. Please use either “bone necrosis” or “bone necrosis.” OR “kienbo*”. This is in the “Topics” field, which includes the title, abstract, “Keyword Plus” and “Author Keyword.” In accordance with the specified criteria, documents categorized as “Articles” were selected, excluding conference abstracts, early access documents, editorial material, corrections, retracted publications, news, book chapters and review articles. Please note that the search was limited to publications up to 2024, since at the time of the search, 2025 was still in progress. The search was conducted on 6 March 2025, in order to avoid changes in the data extracted and the number of studies. A total of 2843 manuscripts were identified in the course of the project.

### 2.2 Study Selection

The metadata of the articles obtained from the search were downloaded into a “.ris” file and imported into the Rayyan web application. Two of the authors then manually reviewed and selected each article based on its title and abstract, aiming to identify those related to osteonecrosis in traumatology and orthopedics. Any discrepancies were resolved by consensus or discussion with a third author. The WOS Accession Number was extracted for records not included in order to exclude them from the initial search, resulting in a total of 1620 publications.

### 2.3 Bibliometric Analysis and Data Standardization

The bibliometric analysis was performed using a previously described methodology [16, 17]. The Bibliometrix package in the R programming language was used to calculate the bibliometric indices. VOSViewer software was used to analyze collaboration networks based on co‐authorship, considering the names of the authors, institutional affiliations, and keywords from the retrieved records. Prior to analysis, the data were manually standardized in the author, institutional affiliation, and keyword fields to eliminate redundancies and inconsistencies. This was achieved by creating thesauri in “.txt” format, adhering to the two‐column scheme (“label” and “replace by”) outlined in the VOSviewer version 1.6.17 software manual (Leiden University, Leiden, Netherlands).

The following metrics were reported: total number of articles; annual variation in production; number of journals; most‐cited articles; journals with the highest number of publications; most‐productive authors; countries with the highest production; affiliations with the greatest impact in the field; co‐authorship networks between authors; co‐occurrence networks according to institutional affiliations; and keyword co‐occurrence networks.

A co‐authorship network analysis was performed using the “fractional counting” method, establishing a minimum of two documents per author or organization. The association normalization method was applied with the following parameters: node repulsion of 4, node attraction of −1, cluster resolution of 1, and a minimum cluster size of 1. Weight was assigned based on the number of articles, and temporality was determined by the average number of publications per year.

Co‐authorship and co‐occurrence networks are represented by nodes, which symbolize the units of analysis involved in the collaboration, and by links reflecting the strength of the relationship based on the number of articles or shared keywords. Node values were defined according to the recommendations in the VOSviewer version 1.6.17 manual.

### 2.4 Ethical Considerations

As this study is an analysis of scientific output in a specific area, it did not require the approval of an ethics committee, as it did not involve contact with humans or animals.

## 3 Results

### 3.1 Publication Output Over Time

**FIGURE 1:**
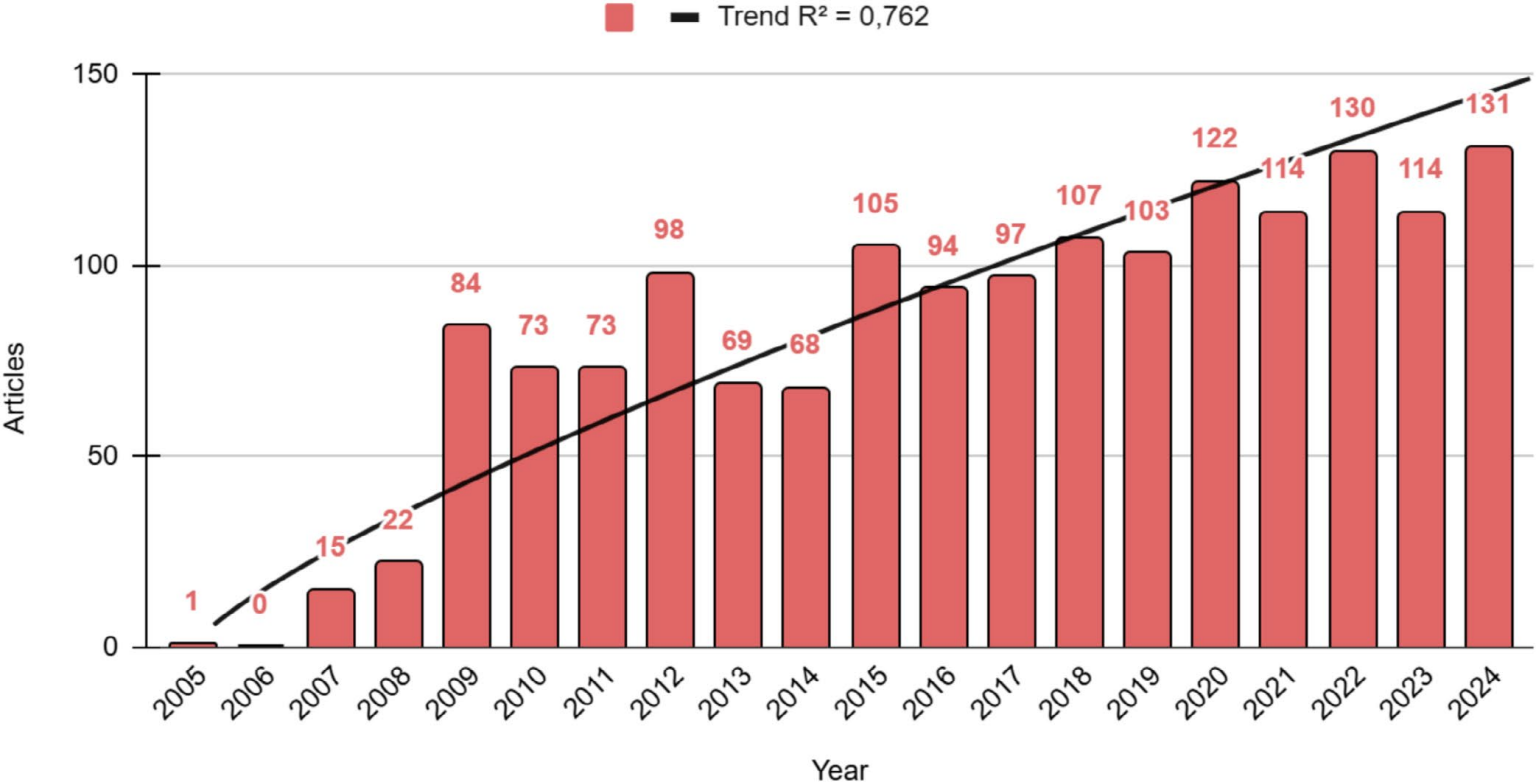
Evolution of the number of articles on osteonecrosis in traumatology and orthopedics.

A total of 1620 publications on scientific output in osteonecrosis in relation to traumatology and orthopedics were found in WOS, published in 78 journals. The first publication was in 2005 and the highest number of publications occurred in 2024 with 131 publications, representing an annual growth rate of 29.25% and an average of 18.72 citations per document (see Figure 1).

### 3.2 Most Productive Authors

**FIGURE 2:**
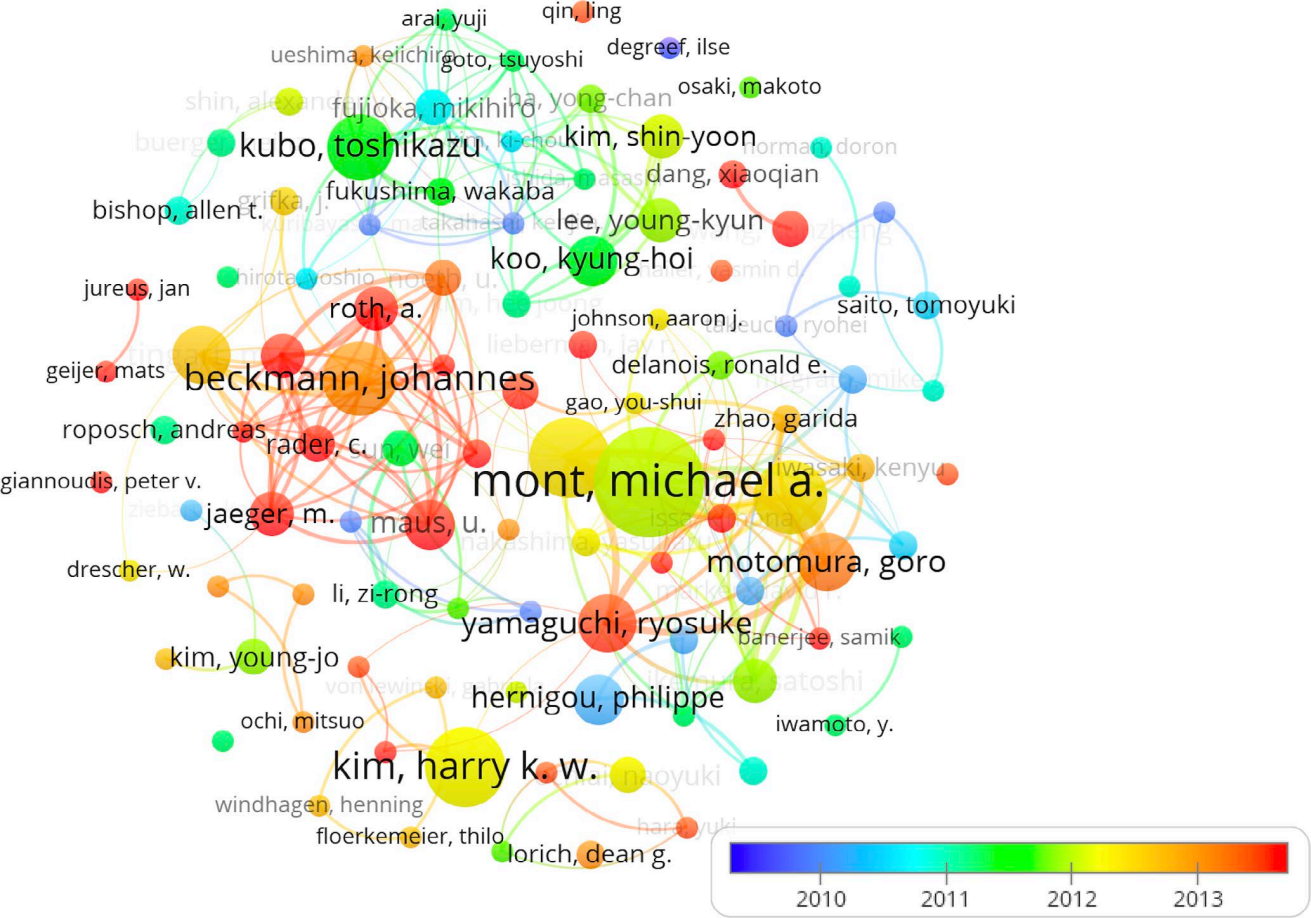
Co‐authorship network in osteonecrosis research in orthopedics and traumatology.

Figure 2 illustrates the most prolific authors in this field: Michael A. Mont, Harry Kwang‐Woo Kim, Philippe Hernigou, and Takuaki Yamamoto. These authors were primarily published between 2011 and 2013. Table 1 presents the 10 authors with the highest scientific output, describing their publication frequency, number of citations, H‐index, and affiliation by country. Michael A. Mont (United States) is in first place with 34 publications, the highest H‐index (86), and 22,813 citations. His most prolific year was 2012, which demonstrates his influence in the field. He is followed by Kim, Hernigou, and Yamamoto (from the United States, France, and Japan, respectively) with 30, 29, and 29 publications each. Their most prolific years were 2012, 2010, and 2011, respectively. Stuart Goodman (United States) has the highest number of citations (23,139) in WOS. Michael A. Mont is second with 22,813 citations, and Yukihide is third with 15,064. Japan is the country with the highest number of authors, accounting for five of the 10 most productive authors.

**TABLE 1:** The 10 most prolific authors in osteonecrosis in the field of orthopedics and traumatology.

N	Author	Frequency	H index in Web of Science	Total publications in Web of Science	Number of citations in Web of Science	Country	Affiliation
1	Michael A. Mont	34	86	1196	22,813	United States	Hospital for Special Surgery
2	Harry Kwang‐Woo Kim	30	36	184	2506	United States	Seoul National University
3	Philippe Hernigou	29	45	243	6070	France	Universite Paris‐Est‐Creteil‐Val‐de‐Marne
4	Takuaki Yamamoto	29	30	136	1925	Japan	Kyushu University
5	Goro Motomura	23	27	16	1605	Japan	Kyushu University
6	Yukihide Iwamoto	21	65	634	15,064	Japan	Kyushu University
7	Yasuharu Nakashima	19	40	494	5602	Japan	Kyushu University
8	Qiushi Wei	19	21	121	1611	China	Guangzhou University of Chinese Medicine
9	Ryosuke Yamaguchi	19	15	56	433	Japan	Kyushu University
10	Stuart Goodman	18	83	658	23,139	United States	Stanford University

### 3.3 Most Cited Articles

The most cited article in the field of osteonecrosis within orthopedics and traumatology was that of Namba et al. [18] in The *Journal of Bone & Joint Surgery*, which received 451 citations and had the highest number of normalized citations (14.22). This was followed by the work of Mont et al. [19] in the same journal, which received 343 citations, giving it an average of 31.18 citations per document. Other highly cited studies include Goran et al. [20] article in *Clinical Biomechanics*, Elsevier, which received 304 citations, and Zalavras et al. [21] article in the *Journal of the American Academy of Orthopaedic Surgeons*, which received 259 citations. Notably, the article with the highest average number of citations per document was that of Zhao et al. [22] in the *Journal of Orthopaedic Translation* (42.17 citations per document), indicating a high relative impact compared to the total number of publications (see Table 2).

**TABLE 2:** The 10 most cited articles in osteonecrosis in the field of orthopedics and traumatology.

N	Title	Author	Year	Journal	DOI	Total citations	Average citations per document	Normalized total citations
1	Risk factors associated with deep surgical site infections after primary total knee arthroplasty: an analysis of 56,216 knees	Namba et al.	2013	The Journal of Bone & Joint Surgery	https://doi.org/10.2106/JBJS.L.00211	451	34.69	14.22
2	Nontraumatic Osteonecrosis of the Femoral Head: Where Do We Stand Today?	Mont et al.	2015	The Journal of Bone & Joint Surgery	https://doi.org/10.2106/JBJS.O.00071	343	31.18	12.47
3	Cortical bone drilling and thermal osteonecrosis	Goran et al.	2012	Clinical Biomechanics Elsevier	https://doi.org/10.1016/j.clinbiomech.2011.10.010	304	21.71	11.36
4	The Natural History of Untreated Asymptomatic Osteonecrosis of the Femoral Head	Mont et al.	2010	The Journal of Bone & Joint Surgery	https://doi.org/10.2106/JBJS.I.00575	300	18.75	9.91
5	Osteonecrosis of the Femoral Head: Evaluation and Treatment	Zalavras et al.	2014	Journal of The American Academy of Orthopaedic Surgeons	https://doi.org/10.5435/JAAOS‐22‐07‐455	259	21.58	8.41
6	Guidelines for clinical diagnosis and treatment of osteonecrosis of the femoral head in adults	Zhao et al.	2020	Journal of Orthopaedic Translation	https://doi.org/10.1016/j.jot.2019.12.004	253	42.17	13.59
7	Nationwide Epidemiologic Survey of Idiopathic Osteonecrosis of the Femoral Head	Fukushima et al.	2018	Clinical Orthopaedics and Related Research	https://doi.org/10.1007/s11999‐010‐1292‐x	245	15.31	8.10
8	Musculoskeletal Consequences of COVID‐19	Disser et al.	2020	The Journal of Bone & Joint Surgery	https://doi.org/10.2106/JBJS.20.00847	244	40.67	13.10
9	Fractures Around the Lateral Cortical Hinge After a Medial Opening‐Wedge High Tibial Osteotomy: A New Classification of Lateral Hinge Fracture	Takeuchi et al.	2012	The Journal of Arthroscopic & Related Surgery	https://doi.org/10.1016/j.arthro.2011.06.034	229	16.36	8.56
10	Surgical Treatment of Three and Four‐Part Proximal Humeral Fractures	Solberg et al.	2009	The Journal of Bone & Joint Surgery	https://doi.org/10.2106/JBJS.H.00133	215	12.65	6.61

### 3.4 Leading Journals and Keyword Trends

**FIGURE 3:**
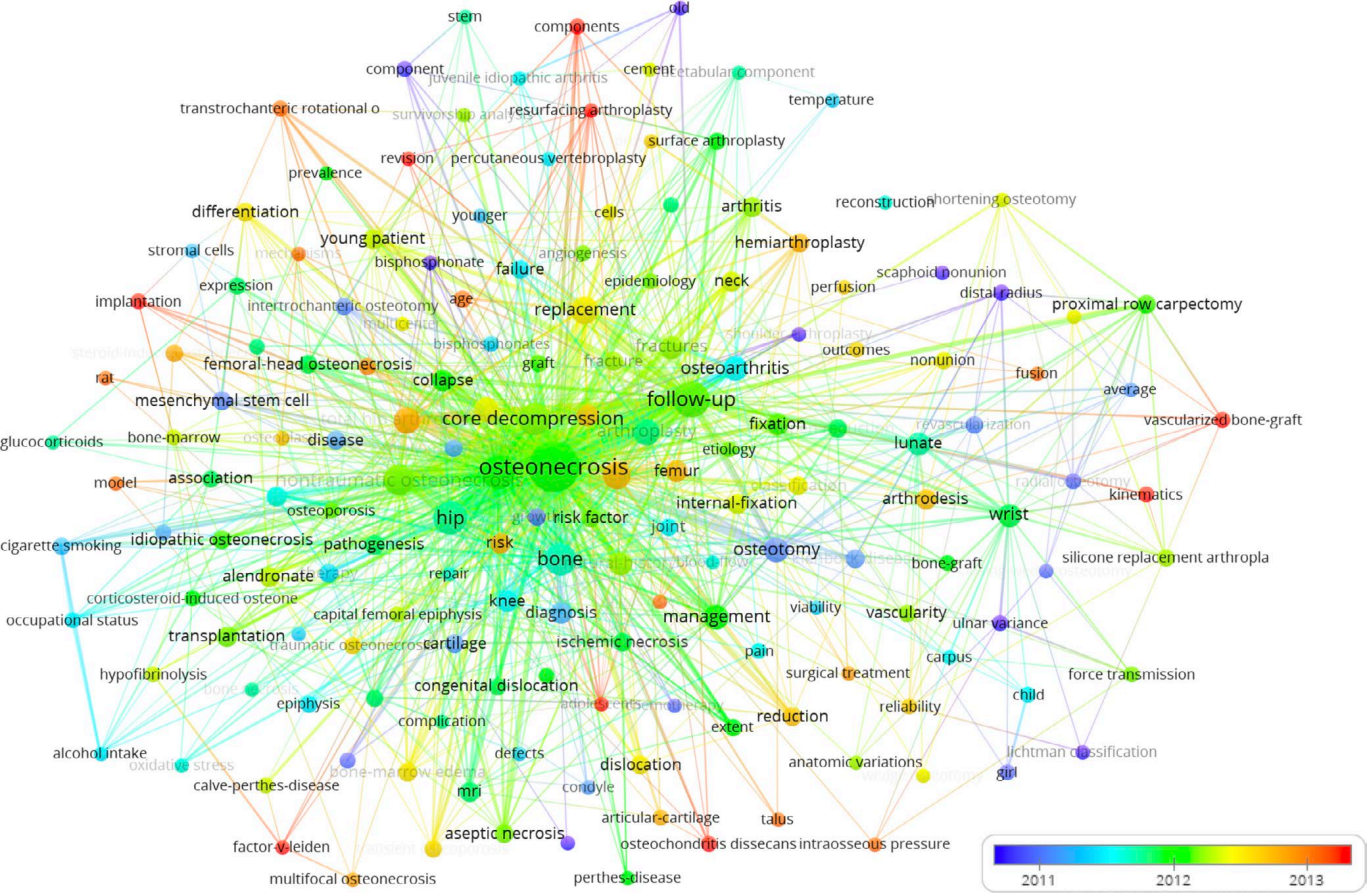
Co‐occurrence of terms relating to osteonecrosis in orthopedics and traumatology.

Table 3 shows the 10 journals with the highest number of publications on osteonecrosis within the fields of orthopedics and traumatology. The journals with the highest number of articles were the *Journal of Orthopaedic Surgery and Research* (414 articles, UK) and *International Orthopaedics* (365 articles, Germany). However, the *Journal of Arthroplasty* (1733 citations, United States) and *International Orthopaedics* (1398 citations) stand out in terms of the number of citations, reflecting their greater impact in the field. Other notable journals include Efort Open Reviews (207 articles, Impact Factor 4.3) and Orthopedic Surgery (199 articles, Australia). The co‐occurrence analysis of terms (Figure 3) revealed that the most frequent and central concepts in the network were “osteonecrosis,” “core decompression,” “follow‐up,” “hip,” and “bone.” These terms are all associated with the predominant therapeutic and anatomical approaches in the field of research. Additionally, the temporal analysis revealed that the most frequently used terms during 2011, were “core decompression,” “ulnar variance,” “old,” “bisphosphonate,” among others; while in 2014 the most common words were “osteochondritis dissecans,” “implantations,” “kinematics,” “vascularized bone graft,” among others.

**TABLE 3:** The 10 journals with the highest number of publications in osteonecrosis in the field of orthopedics and traumatology.

N	Journal	Frequency	Number of cites	JIF	Quartile	Country	Publisher
1	*Journal of Orthopaedic Surgery and Research*	414	291	2.8	1	United Kingdom	BioMed Central Ltd
2	*International Orthopaedics*	365	1398	2	2	Germany	Springer Nature
3	*Journal of Arthroplasty*	272	1733	3,4	1	United States	Elsevier B.V.
4	*Efort Open Reviews*	207	86	4,3	1	United Kingdom	British Editorial Society of Bone and Joint Surgery
5	*Indian Journal of Orthopaedics*	203	155	1,1	3	India	Wolters Kluwer Medknow Publications
6	*Orthopaedic Surgery*	199	171	1,8	2	Australia	Blackwell Publishing Asia Pty Ltd
7	*Foot and Ankle Surgery*	134	165	1,9	2	United Kingdom	Elsevier Ltd
8	*Orthopedics*	126	404	1,1	3	United States	Slack Incorporated
9	*Acta Orthopaedica Belgica*	123	208	0,5	4	Belgium	ARSMB‐KVBMG
10	*Current Reviews in Musculoskeletal Medicine*	123	115	2,9	1	United States	Humana Press

*Note:* The JIF, quartile, country, and publisher were extracted from the 2023 Journal Citation Reports.

Abbreviation: JIF, journal impact factor.

### 3.5 Country‐Level Scientific Production

According to Table 4, China is the world leader in osteonecrosis research in orthopedics and traumatology, having published 375 papers that have been cited 5171 times, giving it an impact factor of 13.79. The United States follows closely behind with 355 publications and the highest citation impact (9578 citations in total and 26.98 citations per document). Japan ranks third with 173 publications and 3361 citations (19.43 per document).

**TABLE 4:** Top 10 countries with the highest number of publications in osteonecrosis in the field of orthopedics and traumatology.

N	Country	Frequency	%	Single‐country publications	Multi‐country publications	Total citations	Average citations per document
1	China	375	27.35	342	33	5171	13.79
2	United States	355	25.89	304	51	9578	26.98
3	Japan	173	12.62	172	1	3361	19.43
4	Germany	124	9.04	106	18	1292	10.42
5	Korea	88	6.42	79	9	1365	15.51
6	France	83	6.05	62	21	1517	18.28
7	United Kingdom	57	4.16	51	6	1391	24.40
8	India	43	3.14	41	2	731	17.00/
9	Turkey	43	3.14	41	2	443	10.30
10	Italy	30	2.19	21	9	734	24.47

## 4 Discussion

This study analyzed the scientific literature on osteonecrosis in traumatology and orthopedics published in the WOS database up to 2024. A total of 1620 publications were identified across 78 journals, showing a steady increase since 2005. The peak in publications occurred in 2024, with 131 articles being published that year. This growth is related to several factors, including the greater recognition of osteonecrosis as a complication of orthopedic procedures, advances in diagnostic techniques favoring early detection of the disease and revolutionary therapeutic measures generating greater worldwide interest [5]. Additionally, ongoing advancements in related medical fields such as cellular and molecular biology, transplantation, pharmacology, and endocrinology have revealed insights into molecular signaling pathways and genetic alterations in the progression of this disease [5].

### 4.1 Influential Authors and Cited Publications

Michael A. Mont, Harry Kwang‐Woo Kim and Philippe Hernigou were the leading researchers with the greatest number of publications in this area. Michael A. Mont is an orthopedic surgeon with over 30 years' experience. He is the editor‐in‐chief of the *Journal of Arthroplasty* and a lifetime member of the Hip Society, the National Osteonecrosis Foundation and the Knee Society. His widely cited publications and the usefulness of his studies for developing clinical guidelines, leading clinical trials, and systematic reviews evaluating the benefits of different osteonecrosis treatments have made him an internationally recognized author [19]. Harry Kwang‐Woo is a pediatric orthopedic surgeon, director of the Centre for Excellence in Hip at Scottish Rite for Children, and a professor at the University of Texas Southwestern Medical Centre [23]. He has described the role of IL‐6 and its modulation in animal models with femoral head osteonecrosis, with the aim of preventing complications and improving bone healing [24]. Additionally, Philippe Hernigou, Director of the Department of Orthopaedic Surgery at Henri Mondor Hospital and Professor at the University of Paris XII, has described cell therapy as a regenerative procedure for lesions caused by osteonecrosis of the femoral head [25]. He found a significant association between the number of transplanted cells and the degree of regeneration [26]. Among authors who have recently published relevant articles, Disser et al. stand out with their 2020 publication, “Musculoskeletal Consequences of SARS‐CoV‐2 Infection,” which discusses possible musculoskeletal complications related to severe viral infections, such as those caused by SARS‐CoV‐2. The article highlights the onset of osteonecrosis associated with high‐dose corticosteroid treatment, a topic of particular relevance in the postpandemic context given the widespread use of these drugs [27]. Finally, Fukushima et al. reported findings such as the peak incidence age of approximately 40 years, hip replacement as the most commonly used therapeutic procedure, followed by osteotomy, and systemic lupus erythematosus as the most prevalent disease among patients with prolonged corticosteroid use, in their 2018 publication “Nationwide Epidemiologic Survey of Idiopathic Osteonecrosis of the Femoral Head” [28].

The most frequently cited publication on osteonecrosis is “Risk Factors Associated with Deep Surgical Site Infections after Primary Total Knee Arthroplasty: An Analysis of 56,216 Knees,” published in *The Journal of Bone and Joint Surgery*. This article addresses topics such as the incidence, risk factors, and management of surgical site infections after total knee arthroplasty. This article is significant in the field due to the patient‐, surgical‐, and hospital‐related risk factors it identifies, such as body mass index, diabetes, cement use, and stays in high‐volume hospitals, all of which are linked to a higher incidence of surgical site infection [18]. Secondly, the article “Nontraumatic osteonecrosis of the femoral head: where do we stand today?” provides an update on concepts regarding osteonecrosis of the femoral head of non‐traumatic etiology. It describes better progression in surgical versus non‐surgical management and shows results based on cell therapy. It also emphasizes the choice of procedures such as total hip arthroplasty in advanced lesions [29]. Finally, the study “Cortical Bone Drilling and Thermal Osteonecrosis” describes the factors involved in heat‐induced bone necrosis in relation to the bone drilling procedure. The study also considers the use of drill guides as a potential source of heat. Furthermore, ultrasound‐guided drilling produced a smoother surface than conventional drilling [30]. These findings demonstrate that osteonecrosis research aims not only to update concepts but also to evaluate the clinical evolution of the disease and improve the early diagnosis and management of its complications in clinical practice.

### 4.2 Journals, Countries, and Research Landscape

The journals with the highest number of publications in the field of orthopedics, traumatology and specifically osteonecrosis include the *Journal of Orthopaedic Surgery and Research*, *International Orthopaedics and the Journal of Arthroplasty*. However, the journal with the highest impact factor is Efort Open Reviews, despite ranking fourth in terms of publication frequency. *Journal of Orthopaedic Surgery*, an open access journal published by BioMed Central, United Kingdom, stands out for its ability to rapidly disseminate both clinical and experimental studies. It has been an important medium for the dissemination of research on the pathophysiology, surgical management and bone regeneration processes in cases of osteonecrosis, especially in Asian regions where this pathology has a high incidence. A representative example is the retrospective study conducted by Zheng et al., which analyzed more than 1100 patients with osteonecrosis of the femoral head and found alterations in lipid and coagulation parameters, highlighting the value of this journal for large‐scale clinical studies [31]. International Orthopaedics, published by Springer Nature, has made a significant contribution to the development of clinical guidelines and systematic reviews on the treatment of osteonecrosis, particularly in the context of hip reconstruction surgery. For instance, the review article by Sultan et al. critically analyzes the main classifications used for osteonecrosis of the femoral head, evaluating their diagnostic utility, reproducibility, and prognostic value [32]. The *Journal of Arthroplasty*, an American journal published by Elsevier B.V., specializes in joint reconstructive surgery, particularly of the hip and knee. The journal's relevance in the field of osteonecrosis lies in its focus on advanced surgical approaches to this pathology, particularly in terminal stages requiring total hip arthroplasty. A representative example is the study by Mitchell et al., which analyzed population data and observed a progressive increase in the use of joint preservation techniques, such as central decompression, demonstrating an evolution in therapeutic decision‐making processes [33]. The journal with the greatest impact is Efort Open Reviews, published by the British Editorial Society of Bone and Joint Surgery in the United Kingdom. It is open access and specializes in high‐quality narrative and systematic reviews that synthesize the available evidence in orthopedics. A relevant article in this journal is that published by Petek et al., which provides a thorough review of the pathophysiological mechanisms leading to femoral head collapse and a critical analysis of the available medical and surgical treatment options [34].

Although China had the highest frequency of research, there are no Chinese journals among the top ten, and only one of the ten most prolific authors is Chinese. This could suggest a lack of appeal or internationalization of these journals. Nevertheless, China is prioritizing and investing in creating its own characteristics and promoting new journals that adapt to current trends, in order to achieve top‐level status [35]. This leadership in production has also been evidenced in recent bibliometric studies. For example, in an analysis of programmed cell death in femoral head osteonecrosis, China accounted for 76.6% of global publications [36]. One of the key reasons for China's interest in orthopedics, particularly osteonecrosis, is the disease's high prevalence in its population, largely due to the widespread use of corticosteroids and alcohol consumption (common risk factors in the region) [37]. Additionally, China's aging population increases the burden of musculoskeletal diseases, justifying heavy investment in the research and development of advanced, joint‐preserving surgical techniques [38]. The combination of clinical need, national health innovation policies, and the pursuit of global scientific leadership has made orthopedics a strategic priority within the science and technology system [39].

We should also bear in mind that the United Kingdom and the United States are among the 10 countries with the highest level of scientific output in the field of osteonecrosis. However, unlike China, which has a high quantity of publications, these countries stand out for having recognized, relevant journals directly linked to osteonecrosis research. Noteworthy journals in the United Kingdom include the *Journal of Orthopaedic Surgery and Research*, EFORT Open Reviews, and Foot and Ankle Surgery. In the United States, notable journals include the Journal of Arthroplasty and Current Reviews in Musculoskeletal Medicine. These results confirm that the use of regional networks in hospitals in the United Kingdom has enabled the development of large databases that contribute to research in this area [40]. The United States also stands out in terms of the number of authors compared to the United Kingdom, which has none. This could be interpreted as indicating greater support or accessibility to publication, given that studies have shown it to have considerable medical and financial resources for research in this area [41].

The high level of interest in this area in both the United Kingdom and the United States can be explained by the significant impact of musculoskeletal disease on their aging populations, the prevalence of risk factors such as chronic corticosteroid use and organ transplantation, and the robust clinical and academic infrastructure available. Furthermore, osteonecrosis is a prevalent cause of premature disability and joint replacement, rendering it a significant health concern in these contexts [42]. For example, in the United States, multiple research studies have been conducted to improve surgical techniques for joint preservation, while the United Kingdom has used multicenter registries to evaluate the evolution and outcomes of these treatments [43]. In contrast, scientific output in regions such as Africa and South America accounts for only 0.98% and 0.66% of total publications, respectively. This is consistent with literature reporting values of 0.4% and 0.2% for countries such as Brazil and South Africa, indicating a minimal increase in scientific output in this field [44].

### 4.3 Thematic Evolution and Clinical Relevance

Keywords reflect the most relevant terms that summarize the main focus of the study. Co‐occurrence analysis is a widely used bibliometric technique that visualizes the content and structure of knowledge on a specific topic, as well as identifying its evolution and emerging topics within the field of orthopedics and traumatology [5]. The most relevant keywords were “mesenchymal stem cell” in 2011, “core decompression” and “follow‐up” in 2012, “femur” in 2013 and “osteochondritis dissecans” in 2014. More recently, clinical terms such as “vascularized bone graft,” “arthrodesis,” have emerged, reflecting a shift toward advanced surgical interventions and regenerative strategies in the therapeutic approach. Today, research on osteonecrosis has evolved from a predominantly clinical and follow‐up focus to an interventionist approach that emphases preserving joint function [45]. This reflects technological advances and growing interest in advanced surgical techniques, such as central decompression and vascularized bone grafting. This reflects technological advances and a growing interest in advanced surgical techniques, such as central decompression and vascularized bone grafting. According to a national US study analyzing 64,739 cases of femoral head osteonecrosis between 2010 and 2020, joint preservation procedures increased, particularly among patients under 50 (15.3%) [33].

Current hotspots in clinical research on osteonecrosis are increasingly centered on joint‐preserving and biologically oriented strategies. In particular, recent trends suggest sustained interest in core decompression, vascularized bone grafting, mesenchymal stem cell‐based approaches, and other hip‐preserving procedures aimed at delaying femoral head collapse and reducing the need for early arthroplasty [33, 44, 45]. This evolution reflects a shift from predominantly descriptive and follow‐up‐based research toward studies focused on earlier intervention, more refined patient selection, and evaluation of functional and structural outcomes after treatment [33, 45]. Taken together, these patterns indicate that contemporary clinical research is moving toward more targeted, regenerative, and outcome‐oriented therapeutic strategies in osteonecrosis.

This suggests a shift in focus from purely observational studies to surgical interventions, which were previously more ethically feasible due to the limited availability of effective techniques and poor understanding of the pathophysiology in the early stages [46]. Now, with greater knowledge of the disease's mechanisms, treatments aimed at preventing joint collapse are prioritized [14]. Vascularized grafting offers an alternative to total arthroplasty and is particularly valuable for young patients for whom prosthetic replacement should be avoided due to its limited lifespan. Furthermore, this option can restore the blood supply, promoting bone regeneration and reducing the necrotic process [47]. Additionally, placing the patient's own mesenchymal stem cells within the damaged bone can open up new regenerative possibilities and has the potential to repair and revitalize ischemic bone. This marks a significant advance in the biological management of osteonecrosis [48].

## 5 Limitations

Although this study presents a rigorous bibliometric analysis, some limitations are inevitable. Firstly, the research was based exclusively on data from the Web of Science Core Collection database, resulting in the exclusion of relevant publications from other data sources. Furthermore, publications from 2025 were not considered due to insufficient time for them to accumulate a representative number of citations. This could have influenced our conclusions, given the speed at which trends and approaches evolve in this field.

## 6 Conclusion

The study of osteonecrosis in orthopedics and traumatology has seen a substantial rise in publications and scientific interest, particularly over the past decade. The leading journals on the subject were identified as the *Journal of Orthopaedic Surgery and Research and International Orthopaedics*, while the *Journal of Arthroplasty* leads in citation impact. China has become the most prolific country in terms of publications on this topic. However, the United States continues to lead in terms of citation impact and author influence, with Michael A. Mont and Harry Kwang‐Woo Kim being particularly notable figures. Thematic analysis revealed a shift toward regenerative medicine and joint preservation, with concepts such as “mesenchymal stem cells” and “vascularised bone graft” playing central roles. These findings are crucial for comprehending global research trends, supporting evidence‐based decision‐making and determining future priorities in the diagnosis and treatment of osteonecrosis.

## Author Contributions

**Guadalupe A. Pomasonco‐Olivas:** writing – original draft, writing – review and editing, formal analysis. **Ulises J. Baldeon‐Necochea:** writing – original draft, writing – review and editing. **Alvaro M. Ñaña‐Cordova:** writing – original draft, writing – review and editing. **Fabriccio J. Visconti‐Lopez:** conceptualization, methodology, visualization, writing – review and editing, writing – original draft.

## Funding

The authors have nothing to report.

## Disclosure

All authors meet the authorship criteria according to the ICMJE guidelines and have approved the final manuscript.

## Conflicts of Interest

The authors declare no conflicts of interest.

## Data Availability

The data that support the findings of this study are available from the corresponding author upon reasonable request.
